# Supplementary material for: Pharmacokinetic Feasibility of Stability-Enhanced Solid-State (SESS) Tenofovir Disoproxil Free Base Crystal
Source: Pharmaceutics. 2023 May 1;15(5):1392. doi: 10.3390/pharmaceutics15051392 (PMC10220743; doi:10.3390/pharmaceutics15051392)
Supplement: Supplementary file 1 [file pharmaceutics-15-01392-s001.zip › pharmaceutics-2343339-supplementary.pdf]

Supplementary Materials: Pharmacokinetic Feasibility of Stability-Enhanced Solid-State (SESS) Tenofovir Disoproxil Free Base Crystal

Byoung Hoon You, Mingoo Bae, Seung Yon Han, Jieun Jung, Kiwon Jung and Young Hee Choi

Title 1: Chromatograms of TEV and IS in rat plasma, urine, and GI samples

Method

The analysis of TEV in biological samples was performed by the modifying the previous reported methods [13–15]. The API 4000 triple quadrupole mass spectrometer system (Framingham, MA, USA) was used for all analyses. The multi reaction monitoring (MRM) mode with an electrospray ionization (ESI) interface was used for positive ions $([M+H])^+$ at a capillary voltage of 5500 V, a desolvation gas temperature of 500 °C, and a nebulizing gas flow of 50 L/min, turbo ion-spray gas flow at 50 L/min, curtain gas flow at 20 L/min, ring voltage at 5500 V, and collision gas (nitrogen) pressure at 5 Torr. The m/z values for TEV and paclitaxel (IS) were 288.330 → 176.100 and 854. 222 → 286.100, respectively. Chromatographic separation was performed using a reversed-phase C_{18} column (Waters X-select C_{18}, 2.1 mm × 100 mm i.d., 3.5 μm particle size; Waters) at a flow rate of 0.4 mL/min. The mobile phase was composed of 0.1% formic acid in water (A) and 0.1% formic acid in acetonitrile (B). The gradient elution was performed using the mobile phase with the following ratio of A:B at 90:10 (v/v) initially, was hanged to a ratio of 10:90 (v/v) at 3.5 min, and was returned to the initial composition at 6.1 min, which was then maintained for 7 min. The analytical data were processed using Analyst software (AB Sciex, CA, USA).

To prepare working solutions and biological standard samples of TEV, 20 mg/mL of stock solutions of TEV in methanol were serially diluted. The working solution of TEV was added to drug-free plasma samples to achieve final concentrations of 0.01, 0.025, 0.1, 0.5, 1, 2, 5, 10, 20, or 200 μg/mL. To deproteinize a 50 μL biological sample, 200 μL of acetonitrile containing 1 μg/mL of IS was added. The sample was vortexed, centrifuged for 10 min at 13,000 rpm, and 10 μL of the supernatant was injected into the column.

Result

The retention times of TEV chromatogram were 0.92 min, and peak shape was symetric. The TEV in blank plasma, urine, and GI was detected without interference, and detection limits of TEV in biological standard samples of plasma, urine, and GI samples were 0.01, 1, and 0.1 μg/mL, respectively. The ragnes of calibration curves for TEV in biological standard samples of plasma, urine, and GI were 0.01–1 μg/mL, 1–20 μg/mL, and 0.1–20 μg/mL, respectively obtained from their peak area ratios relative to those of the IS by linear regression.

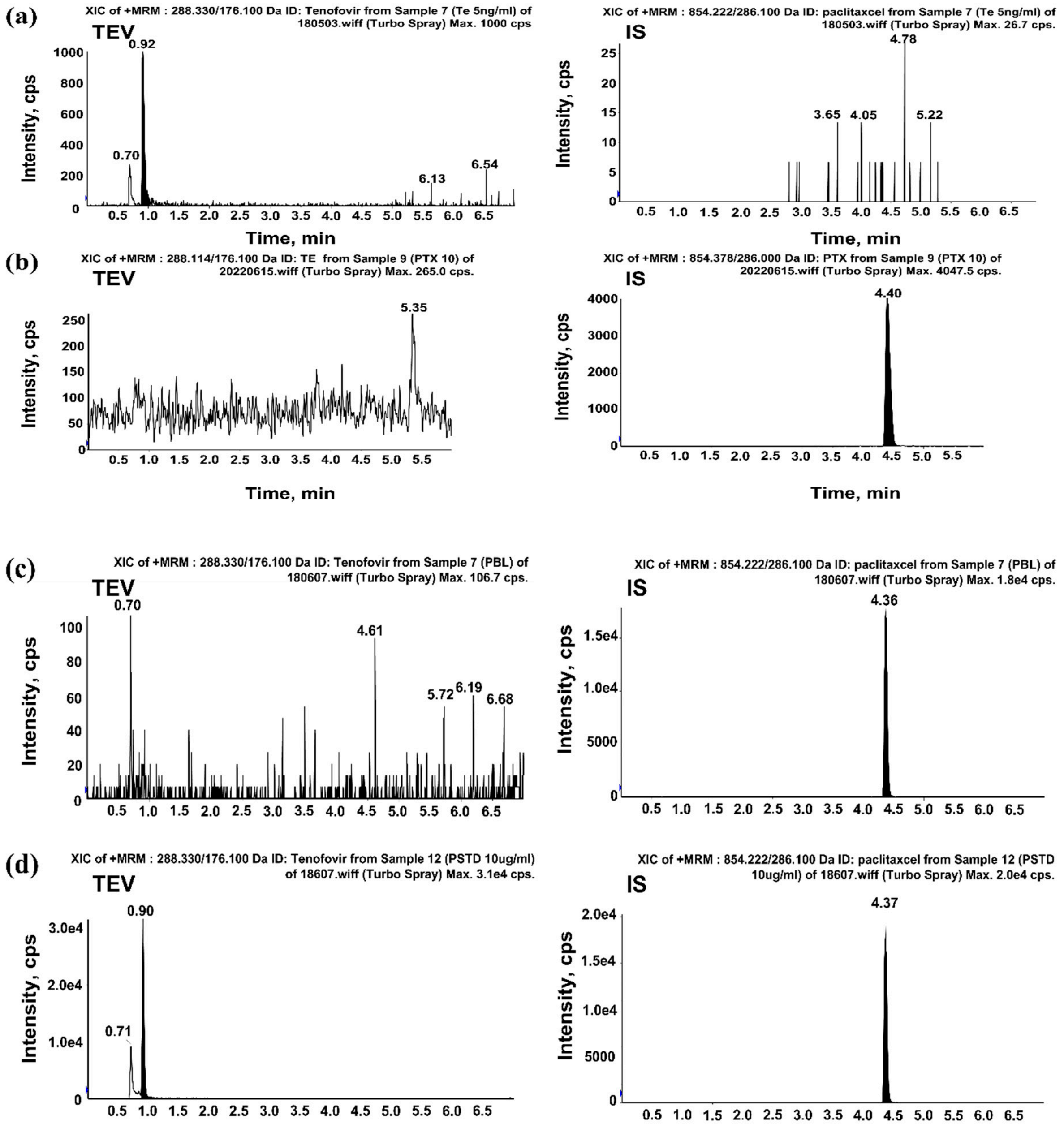
(a)
XIC of +MRM : 288.330/176.100 Da ID: Tenofovir from Sample 7 (Te 5ng/ml) of 180503.wiff (Turbo Spray) Max. 1000 cps
TEV
XIC of +MRM : 854.222/286.100 Da ID: paclitaxcel from Sample 7 (Te 5ng/ml) of 180503.wiff (Turbo Spray) Max. 26.7 cps.
IS
Intensity, cps
Time, min
(b)
XIC of +MRM : 288.114/176.100 Da ID: TE from Sample 9 (PTX 10) of 20220615.wiff (Turbo Spray) Max. 265.0 cps.
TEV
XIC of +MRM : 854.378/286.000 Da ID: PTX from Sample 9 (PTX 10) of 20220615.wiff (Turbo Spray) Max. 4047.5 cps.
IS
(c)
XIC of +MRM : 288.330/176.100 Da ID: Tenofovir from Sample 7 (PBL) of 180607.wiff (Turbo Spray) Max. 106.7 cps.
TEV
XIC of +MRM : 854.222/286.100 Da ID: paclitaxcel from Sample 7 (PBL) of 180607.wiff (Turbo Spray) Max. 1.8e4 cps.
IS
(d)
XIC of +MRM : 288.330/176.100 Da ID: Tenofovir from Sample 12 (PSTD 10ug/ml) of 18607.wiff (Turbo Spray) Max. 3.1e4 cps.
TEV
XIC of +MRM : 854.222/286.100 Da ID: paclitaxcel from Sample 12 (PSTD 10ug/ml) of 18607.wiff (Turbo Spray) Max. 2.0e4 cps.
IS

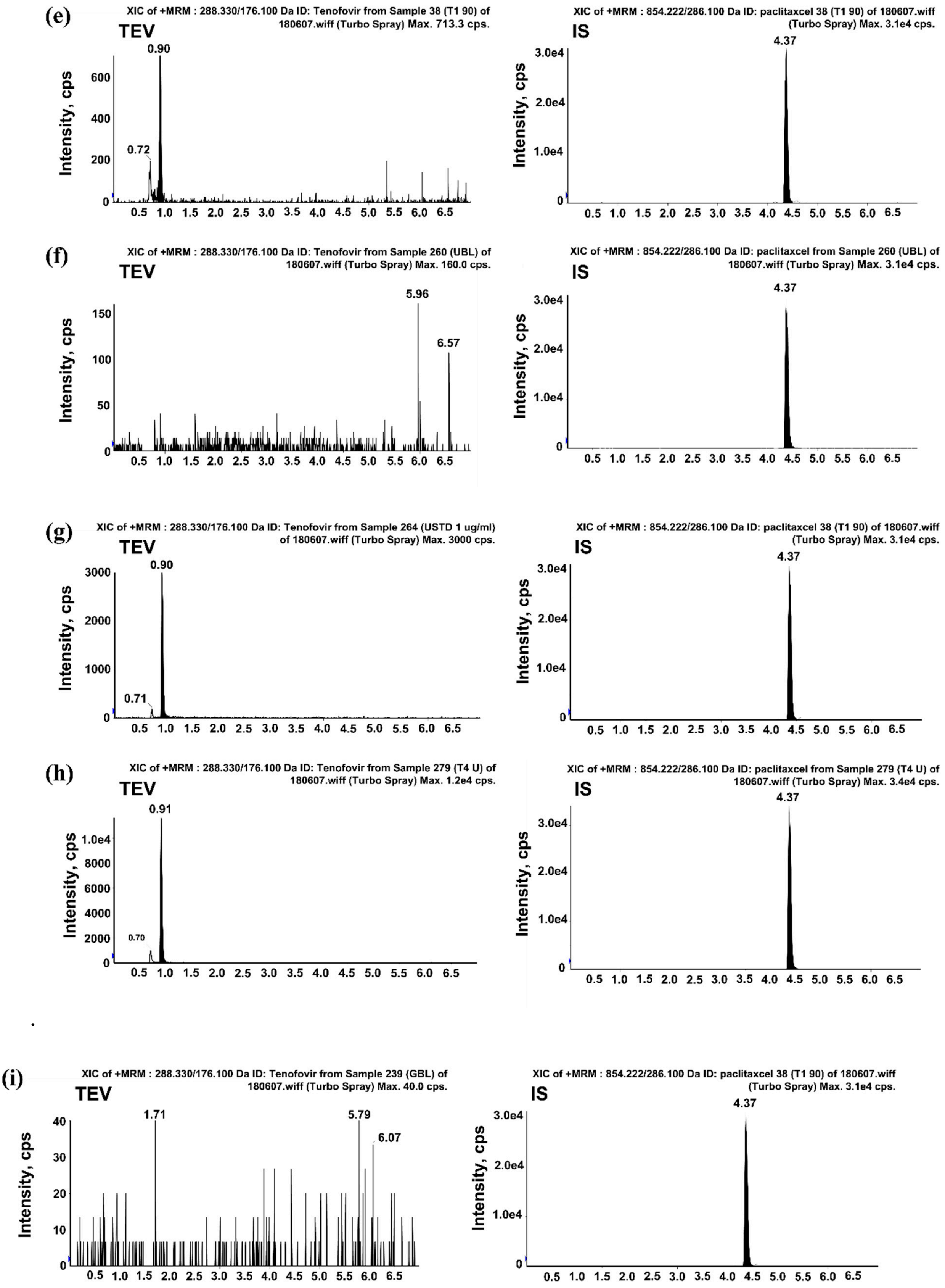

(e)
XIC of +MRM : 288.330/176.100 Da ID: Tenofovir from Sample 38 (T1 90) of 180607.wiff (Turbo Spray) Max. 713.3 cps.
TEV
Intensity, cps
0.90
0.72
XIC of +MRM : 854.222/286.100 Da ID: paclitaxcel 38 (T1 90) of 180607.wiff (Turbo Spray) Max. 3.1e4 cps.
IS
4.37
(f)
XIC of +MRM : 288.330/176.100 Da ID: Tenofovir from Sample 260 (UBL) of 180607.wiff (Turbo Spray) Max. 160.0 cps.
TEV
5.96
6.57
XIC of +MRM : 854.222/286.100 Da ID: paclitaxcel from Sample 260 (UBL) of 180607.wiff (Turbo Spray) Max. 3.1e4 cps.
IS
4.37
(g)
XIC of +MRM : 288.330/176.100 Da ID: Tenofovir from Sample 264 (USTD 1 ug/ml) of 180607.wiff (Turbo Spray) Max. 3000 cps.
TEV
0.90
0.71
XIC of +MRM : 854.222/286.100 Da ID: paclitaxcel 38 (T1 90) of 180607.wiff (Turbo Spray) Max. 3.1e4 cps.
IS
4.37
(h)
XIC of +MRM : 288.330/176.100 Da ID: Tenofovir from Sample 279 (T4 U) of 180607.wiff (Turbo Spray) Max. 1.2e4 cps.
TEV
0.91
0.70
XIC of +MRM : 854.222/286.100 Da ID: paclitaxcel from Sample 279 (T4 U) of 180607.wiff (Turbo Spray) Max. 3.4e4 cps.
IS
4.37
(i)
XIC of +MRM : 288.330/176.100 Da ID: Tenofovir from Sample 239 (GBL) of 180607.wiff (Turbo Spray) Max. 40.0 cps.
TEV
1.71
5.79
6.07
XIC of +MRM : 854.222/286.100 Da ID: paclitaxcel 38 (T1 90) of 180607.wiff (Turbo Spray) Max. 3.1e4 cps.
IS
4.37

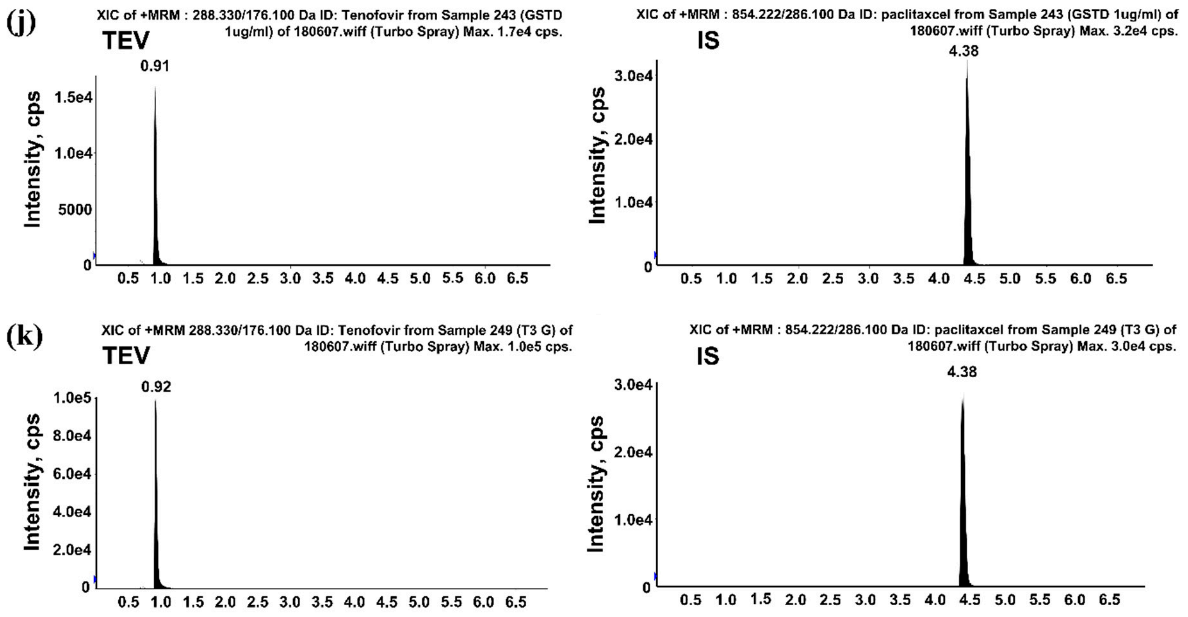

Figure S1. Representative chromatograms of TEV and IS in rat plasma, urine, and GI samples. (a) Stock solution of 5 ng/mL TEV; (b) Stock solution of 10 ng/mL paclitaxel (IS); (c) drug-free rat plasma; (d) plasma standard spiked with 10 μg/mL TEV; (e) plasma sample at 90 min after oral administration of 20 mg/kg TEV; (f) drug-free urine; (g) urine standard spiked with 1 μg/mL TEV; (h) urine sample collected at 24 h after oral administration of 20 mg/kg TEV; (i) drug-free GI; (j) GI standard spiked with 1 μg/mL TEV; (k) GI sample collected at 24 h after oral administration of 20 mg/kg TEV. The structures of TEV and IS are presented.

Title 2: Conversions of SESS-TD crystal or TDF to TEV in rat plasma

Method

To determine whether the stability-enhanced solid state TD free base crystal (SESS-TD crystal) or tenofovir disoproxil fumarate (TDF) converted to tenofovir (TEV) in rat plasma, 1 mL of rat plasma was spiked with 10 μL of SESS-TD crystal or TDF to a final concentration of 0.1 μg/mL as TEV. At 0, 30, 60, and 120 min after spiking, a 50 μL of rat plasma containing SESS-TD crystal or TDF was transferred to the microtube. To deprotenize the sample, 100 μL acetonitrile containing 0.05 μg/mL of carbamazepine (as IS) was added to the tube. After vortexing and centrifusing for 10 min at 16,060 g, the supernatant was collected. The concentrations of TEV and TD were analyzed by LC-MS/MS as desribed below.

The analysis of TEV and TD in SESS-TD crystal and TDF in rat plasma was performed by using the API 4000 triple quadrupole mass spectrometer system (Framingham, MA, USA). The multi reaction monitoring (MRM) mode with an electrospray ionization (ESI) interface was used for positive ions ($[M+H])^+$ at a capillary voltage of 5500 V, a desolvation gas temperature of 500 °C, and a nebulizing gas flow of 50 L/min, turbo ion-spray gas flow at 50 L/min, curtain gas flow at 20 L/min, ring voltage at 5500 V, and collision gas (nitrogen) pressure at 5 Torr. The *m/z* values for TEV, TD, and IS were 288.149 → 159.000, 520.200 → 270.100, and 237. 241 → 194.300, respectively. Chromatographic separation was performed using a reversed-phase C_{18} column (Waters X-select C_{18}, 2.1

mm × 100 mm i.d., 3.5 μm particle size; Waters) at a flow rate of 0.4 mL/min. The mobile phase was composed of 0.1% formic acid in water (A) and 0.1% formic acid in acetonitrile (B). The gradient elution was performed using the mobile phase with the following ratio of A:B at 90:10 (*v*/*v*) initially, was changed to a ratio of 10:90 (*v*/*v*) at 5 min, and was returned to the initial composition at 6.51 min, which was then maintained for 9 min. The analytical data were processed using Analyst software (AB Sciex, CA, USA).

In rat plasma samples spiked with SESS-TD crystal or TDF, the changes in TD or TEV concentration over time were expressed as the the percentage of TD or TEV concentration compared to that at 0 min, respectively.

A p value < 0.05 was deemed to be statistically significant using a Student's t-test between the two means for the unpaired data.

Result

As shown in Figure S2, in rat plasma spiked with SESS-TD crystal, the percentages of remaining TD concentrations at 30, 60, and 120 min were 35.2 ± 15.1%, 15.6 ± 12.1%, and 11.9 ± 4.93%, respectively, compared to 0 min. In rat plasma spiked with TDF, the percentages of remaining TD concentrations at 30, 60, and 120 min were 26.3 ± 27.7%, 8.26 ± 0.693%, and 3.73 ± 1.36%, respectively, compared to 0 min.

In rat plasma spiked with SESS-TD crystal, the changed percentages of TEV concentrations at 30, 60, and 120 min were 81.7 ± 38.1%, 104 ± 25.6%, and 142 ± 41.4%, respectively, compared to 0 min. In rat plasma spiked with TDF, the changed percentages of TEV concentrations at 30, 60, and 120 min were 134 ± 54.0%, 155 ± 4.36%, and 181 ± 43.0%, respectively, compared to 0 min. As expectedly, the peaks of TEV were observed at 120 min after spiking SESS-TD crystal or TDF in rat plasma, indicating that TD in SESS-TD crystal or TDF was converted to TEV. The conversion of SESS-TD crystal to TEV showed a similar pattern as the case of TDF.

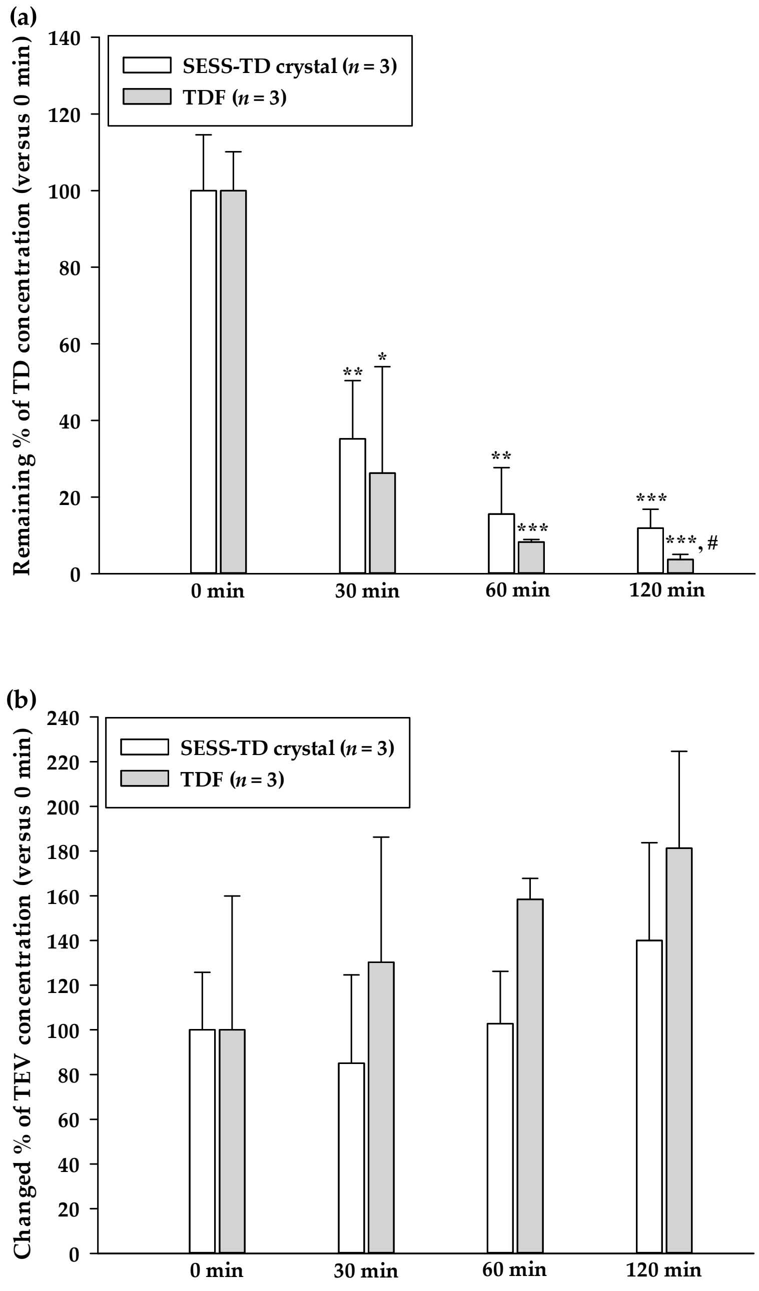

Figure S2. (a) The remaining percentage (%) of TD concentration over time compared to that at 0 min in rats plasma spiked with SESS-TD crystal or TDF. (b) The changed percentage (%) of TEV concentration over time compared to that at 0 min in rat plasma spiked with SESS-TD or TDF. *Significantly different ($p < 0.05$) from 0 min. **Significantly different ($p < 0.01$) from 0 min. ***Significantly different ($p < 0.001$) from 0 min. #TDF group was significantly different ($p < 0.05$) from SESS-TD crystal group at the same sampling time.

Table S1. Peak areas of TEV, TD, and IS in LC-MS/MS analysis of rats plasma spiked with SESS-TD crystal and TDF. Each sample was obtained at 0, 30, 60, and 120 min after the spiking SESS-TD crystal or TDF.

Sample name	Sample No.	TD (area)	TEV (area)	IS (area)
SESS-TD crystal at 0 min	1	2.88e+5	3.02e+3	9.77e+5
	2	3.86e+5	1.85e+3	1.02e+6
	3	3.62e+5	2.45e+3	9.32e+5
SESS-TD crystal at 30 min	1	1.94e+5	1.05e+3	1.04e+6
	2	9.55e+4	3.00e+3	1.04e+6
	3	1.14e+5	2.69e+3	1.19e+6
SESS-TD crystal at 60 min	1	1.08e+5	2.14e+3	1.04e+6
	2	2.57e+4	2.71e+3	1.10e+6
	3	4.96e+4	4.29e+3	1.24e+6
SESS-TD crystal at 120 min	1	6.36e+4	4.84e+3	1.03e+6
	2	3.90e+4	3.52e+3	1.06e+6
	3	3.54e+4	3.38e+3	1.32e+6
TDF at 0 min	1	2.08e+5	3.11e+3	9.77e+5
	2	2.89e+5	1.22e+3	1.02e+6
	3	3.12e+5	1.87e+3	1.20e+6
TDF at 30 min	1	1.47e+5	3.95e+3	1.04e+6
	2	2.82e+4	2.02e+3	1.04e+6
	3	3.26e+4	2.92e+3	1.22e+6
TDF at 60 min	1	2.37e+4	3.33e+3	1.04e+6
	2	2.00e+4	3.17e+3	1.10e+6
	3	2.75e+4	4.27e+3	1.27e+6
TDF at 120 min	1	1.38e+4	4.97e+3	1.03e+6
	2	9.83e+3	3.53e+3	1.06e+6
	3	8.39e+3	4.21e+3	1.28e+6
